# Undergraduate ophthalmology education according to International Council of Ophthalmology guidelines: A systematic review

**DOI:** 10.3205/zma001753

**Published:** 2025-04-15

**Authors:** Luksanaporn Krungkraipetch, Naruporn Krungkraipetch, Gamon Savatsomboon

**Affiliations:** 1 Burapha University, Faculty of Medicine, Department of Ophthalmology, Chonburi, Thailand; 2 Phaholpolpayuhasena Hospital, Kanchanaburi District, Kanchanaburi, Thailand; 3 Mahasarakham University, Faculty member of MBS, Mahasarakham, Thailand

**Keywords:** medical education, ophthalmology, undergraduate, International Council of Ophthalmology

## Abstract

**Objectives:**

The research focused on the recommendations of the International Council of Ophthalmology, specifically regarding the duration of ophthalmology education, areas of clinical exposure, essential skills, and adherence to these standards internationally.

**Methodology:**

A thorough search was conducted in the PubMed, Cochrane Library, Scopus, and ERIC databases up to April 2024 to identify studies related to International Council of Ophthalmology (ICO) principles in undergraduate medical education. Two independent reviewers assessed citations for inclusion criteria, gathered data, and evaluated the risk of bias using the ROBIN-I tool, PROSPERO CRD42024517718.

**Findings:**

From 537 unique references, only eight research articles qualified for inclusion. The primary educational aim in most studies was to meet the ICO requirements. Typically, medical students spend at least two weeks focusing on ophthalmology. These courses offer extensive exposure to ophthalmic patients across diverse clinical environments, such as ophthalmology clinics, emergency departments, and surgical theaters. Instructional methods include theoretical lectures, small group discussions, self-directed learning, and hands-on clinical experiences. The study found that the adoption of ICO recommendations varied from 20% to 36%.

**Conclusions:**

This research evaluates how undergraduate ophthalmology education in medical schools correlates with ICO guidelines, indicating that implementation remains limited. Enhanced promotion of these standards in educational institutions is essential, alongside further studies.

## Zusammenfassung

**Zielsetzungen:**

Die Forschung konzentrierte sich auf die Empfehlungen des Internationalen Rates für Ophthalmonolgie (International Council of Ophthalmology, kurz: ICO), insbesondere in Bezug auf die Dauer der Ausbildung in der Augenheilkunde, die Bereiche der klinischen Exposition, die wesentlichen Fähigkeiten und die Einhaltung dieser Standards auf internationaler Ebene.

**Methodik:**

Es wurde eine gründliche Suche in den Datenbanken PubMed, Cochrane Library, Scopus und ERIC bis April 2024 durchgeführt, um Studien zu identifizieren, die sich auf die Grundsätze des ICO in der medizinischen Grundausbildung beziehen. Zwei unabhängige Gutachter bewerteten die Zitate nach Einschlusskriterien, sammelten Daten und bewerteten das Risiko einer Verzerrung mit dem ROBIN-I-Tool, PROSPERO CRD42024517718.

**Ergebnisse:**

Von 537 Einzelreferenzen qualifizierten sich nur acht Forschungsartikel für die Aufnahme. Das primäre Ausbildungsziel der meisten Studien bestand darin, die ICO-Anforderungen zu erfüllen. In der Regel verbringen Medizinstudenten mindestens zwei Wochen mit dem Schwerpunkt Augenheilkunde. Diese Kurse bieten einen umfassenden Umgang mit Augenpatienten in verschiedenen klinischen Umgebungen, z. B. in Augenkliniken, Notaufnahmen und Operationssälen. Zu den Lehrmethoden gehören theoretische Vorlesungen, Diskussionen in kleinen Gruppen, selbstgesteuertes Lernen und praktische klinische Erfahrungen. Die Studie ergab, dass die Übernahme der ICO-Empfehlungen zwischen 20% und 36% lag.

**Schlussfolgerungen:**

Diese Studie bewertet, wie die Ophthalmologieausbildung an medizinischen Fakultäten mit den ICO-Leitlinien korreliert, und zeigt, dass die Umsetzung noch begrenzt ist. Eine verstärkte Förderung dieser Standards in den akademischen Einrichtungen ist neben weiteren Studien unerlässlich.

## Introduction

Vision impairment represents a critical health issue. Worldwide, approximately 2.2 billion individuals are affected by visual impairment, with over 1 billion suffering from moderate to severe vision loss or blindness due to conditions that could be prevented or treated [1]. Therefore, understanding eye diseases is essential for meeting the requirements.

Over the years, medical education experts have debated the inclusion of ophthalmology in undergraduate studies, its role in the medical curriculum, and the teaching methods used for this subject [2]. Primary care and specialty physicians must have a strong understanding of basic ophthalmology principles and skills. This knowledge is essential because eye issues frequently arise among patients visiting ambulatory healthcare providers [3], [4]. The International Council of Ophthalmology (ICO) prioritizes ophthalmology education as a vital part of its strategy to preserve and restore vision worldwide. The ICO guides medical students, equipping them to become primary care physicians capable of diagnosing and treating eye conditions. In 1999, the global ophthalmology strategic plan aimed at vision preservation led to an international task force focused on ophthalmic medical education.

In 2006, the ICO recommended specific teaching methods, such as lectures, clinical demonstrations, case studies, and evidence-based medicine education. This approach combines ophthalmic education with neuroscience, neurology, endocrinology, and geriatric medicine. It is advised that all medical schools include an evidence-based ophthalmology curriculum as part of their core subjects. The curriculum components are organized by the time dedicated to the curriculum, specific teaching methods, and the resources and materials outlined in the report. Forty to sixty hours, equivalent to 5-8 days, is suggested for this study. The content can be divided into 12 focus areas, which cover the “fundamentals of ophthalmology, cornea and external diseases, lens and cataract, neuro-ophthalmology, vitreoretinal disease, glaucoma, pediatric ophthalmology and strabismus, diseases of the eyelid, lacrimal system and orbit, ocular manifestations of systemic disease, intraocular tumors, refraction, and contact lenses, and refractive surgery” [5].

Currently, ophthalmology education worldwide lacks adherence to standardized criteria. Therefore, the researchers sought to explore how ophthalmology studies are organized across various medical schools. This systematic review aims to evaluate the state of undergraduate ophthalmology education concerning ICO recommendations: the duration of ophthalmology training in medical school, areas of clinical exposure, fundamental ophthalmology skills, and the degree of compliance with ICO standards implemented.

## Methods

We extensively assessed the Preferred Reporting Items for Systematic Reviews and Meta-Analyses (PRISMA) criteria, detailed in the enclosed attachment 1 , supplementary material 1 [6]. The International Prospective Register of Systematic Reviews (PROSPERO) has recognized our protocol, registration CRD42024517718.

### Data sources and searches

In April 2024, we extensively searched the PubMed, Cochrane CENTRAL, ERIC, and Scopus databases for publications from 2000 to 2024, as outlined in attachment 1 , supplementary material 2. The search strategies employed a combination of unstructured language and subject headings pertinent to the databases, highlighting key aspects of medical education within ophthalmology, particularly undergraduates and the International Council of Ophthalmology, where possible. A deliberate attempt was made to ensure a comprehensive and sensitive search to optimize the output. We manually reviewed the reference lists from studies that fulfilled the inclusion criteria and the articles featured in the reviews discovered during our database search. This was done to find any additional relevant studies.

### Study selection

The procedure began by importing all database citations into EndNote version 20. This software identifies and resolves potential duplicate entries by comparing titles, authors, journals, and publication years. After using EndNote to detect duplicates, LK manually removed the identified duplicate records.

A study was considered eligible for inclusion if it met these criteria:

1. Participants included medical schools, students, interns, and first-year residents.
2. The intervention discussed pertains to the recommendations of the International Council of Ophthalmology. Studies that did not comply with the ICO requirements were excluded, regardless of their design (see attachment 1 , supplementary material 2).

With this search methodology, two reviewers (LK and NK) independently evaluated the titles and abstracts of all retrieved articles. Any conflicts arising during the screening process were resolved by consensus, and papers that passed the screening criteria were chosen for comprehensive text analysis. LK and NK reviewed the full text based on specific inclusion/exclusion criteria. All disagreements at this stage were resolved through discussion until a consensus was reached.

### Data extraction and risk of bias assessment

A data extraction form was created to collect specific data points from each included study. Two reviewers, LK and NK, independently gathered qualitative and quantitative data from all references. This data encompassed research design, participant counts and characteristics, study goals, measurement techniques, findings, and a comprehensive quality assessment of the studies.

Two authors, NK and GS, independently assessed the bias likelihood for each quantitative study using the likelihood tool. The ROBINS-I tool is utilized to evaluate bias in nonrandomized therapy studies. The authors classified the overall risk of bias into seven distinct categories: low, moderate, severe, or critical. A third author (LK) assessed any discrepancies in the bias evaluation (see attachment 1 , supplementary material 3).

### Data synthesis and statistical analysis

The study explored the ICO standards established for undergraduate medical education. These standards included the length of ophthalmology instruction, key areas of clinical exposure, and essential clinical tasks. The percentage of ICO guidelines utilized in medical curricula was used to evaluate how ICO guidelines were integrated into medical curricula.

No meta-analyses were performed due to the diversity of study designs, outcomes, and metrics. Statistical analysis using R determined the prevalence of ICO guidelines in medical curricula and the ICO core clinical exposure areas and competencies program.

## Results

### Search results

**Figure 1:**
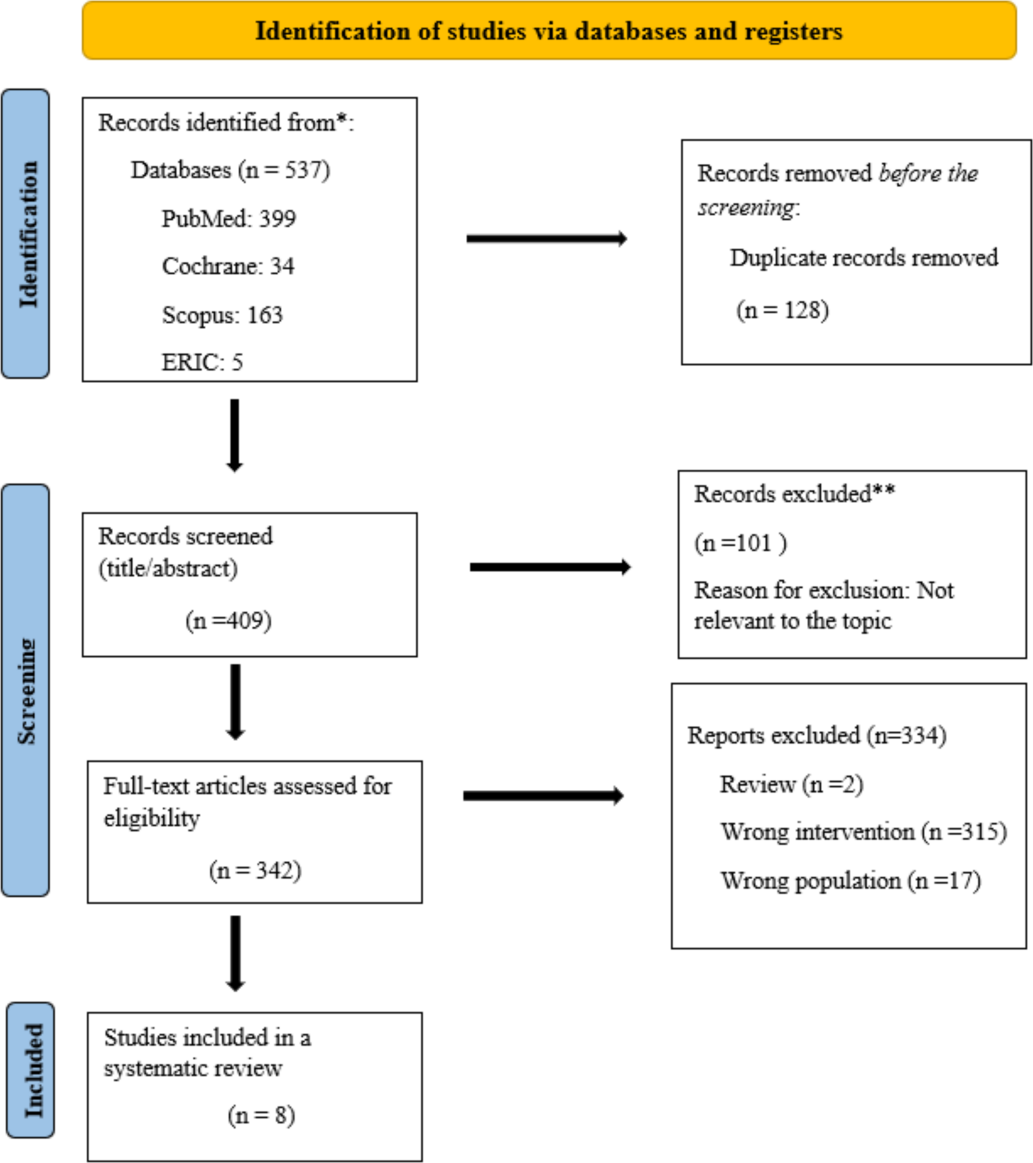
The PRISMA flow diagram

The PRISMA flow diagram shown in figure 1 (Fig. 1) illustrates the search process. Our electronic database search resulted in 537 citations. After eliminating 128 duplicates, we had 409 unique citations for further evaluation. Of these, 342 publications were screened, and only 11 research papers met our qualifying criteria. After carefully reviewing the references from the two articles excluded in the full text review stage, we opted to add one more study. As a result, we included eight studies in the final qualitative analysis.

### Characteristics of the study

Table 1 (Tab. 1) presents the study’s characteristics. Each research effort employed a cross-sectional design featuring a sample size of between 8 and 15 participants. The investigations were conducted in several countries, including Canada [7], [8], Saudi Arabia [9], [10], the United Kingdom [11], Australia [12], India [13], and Nigeria [14]; 37.5% (n=3) were recorded at medical schools [8], [11], [12], 37.5% (n=3) among medical students [9], [10], [13], in one medical intern (n=1) [14], and one first-year resident (n=1). The study sample comprised 96 medical schools and 1,288 individuals, including medical students, medical interns, and first-year residents. The primary objective of most research is to evaluate the extent to which undergraduate ophthalmology education aligns with the recommendations set forth by the ICO, with a success rate of 62.5% [7], [9], [10], [11], [14]. All research utilized questionnaire measurement, with five out of eight investigations employing an online questionnaire [7], [8], [9], [11], [12].

### Bias risk

**Figure 2:**
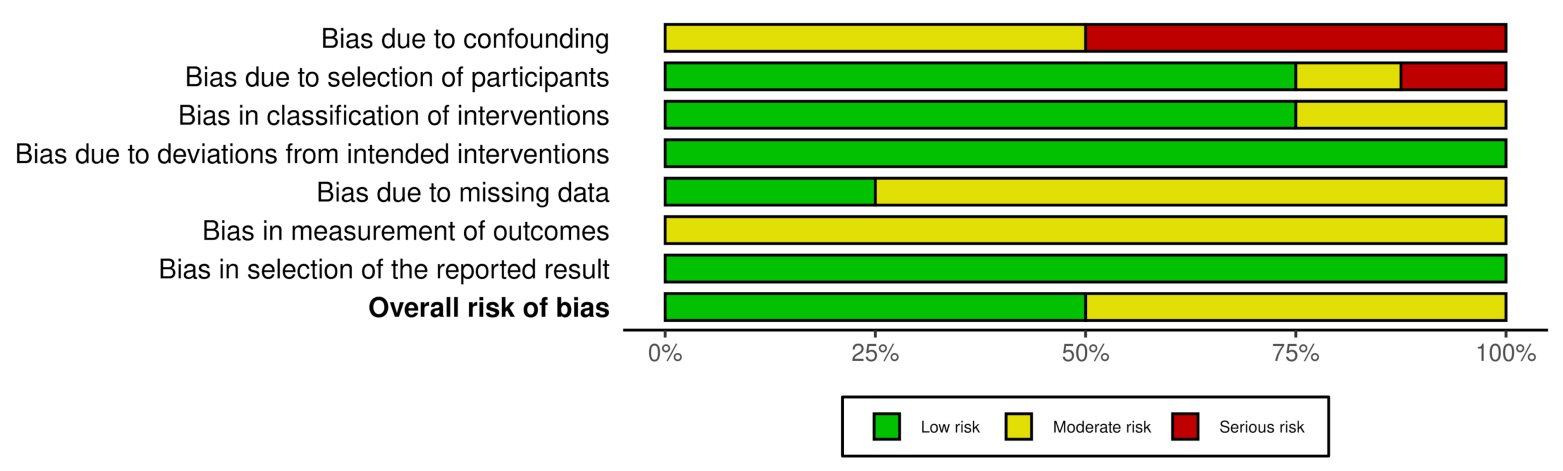
Summary of the bias risk

**Figure 3:**
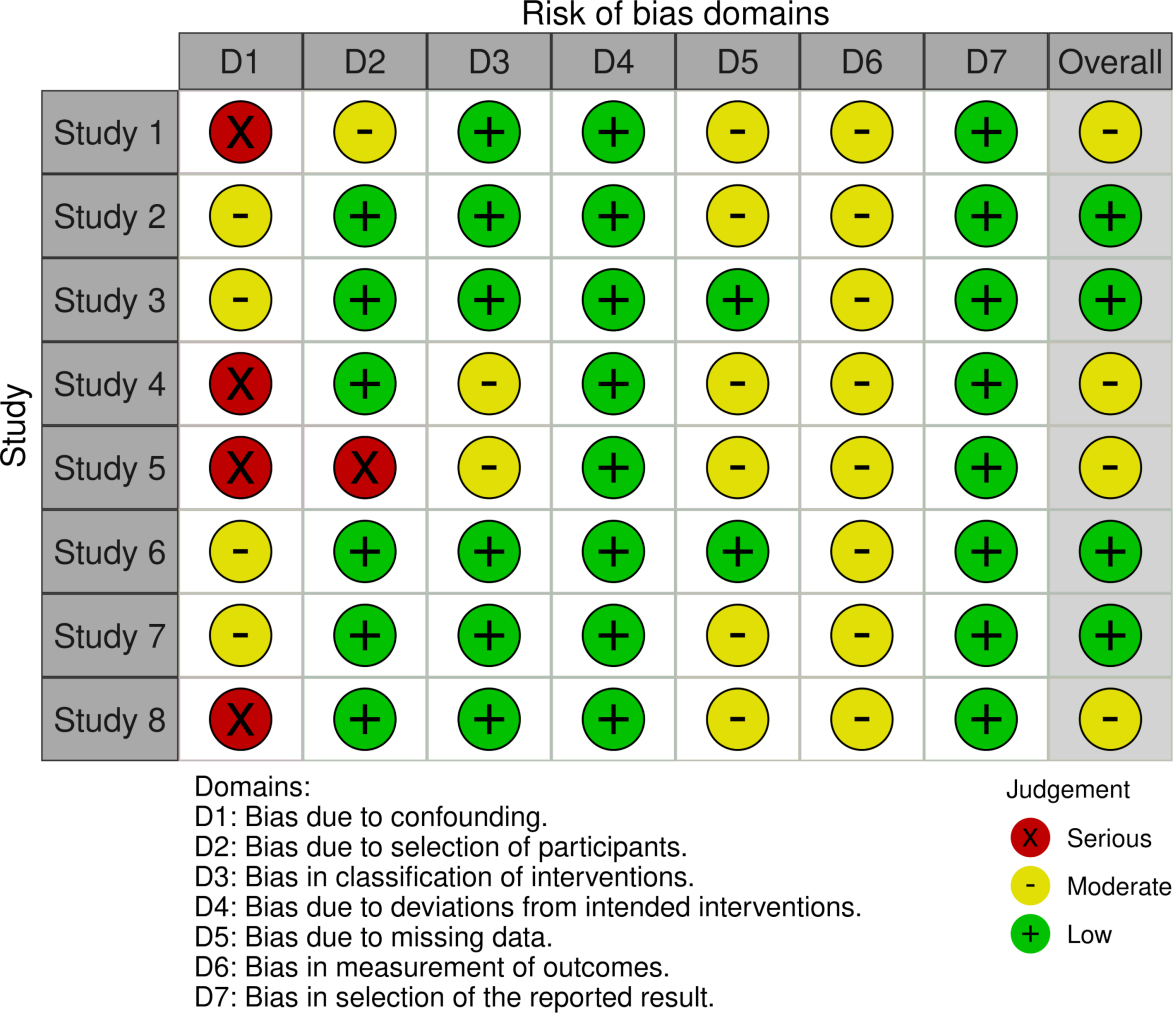
Traffic light diagram of the bias risk ROBINS-I

The overall risk of bias across various domains was evaluated as moderate. The associated risk levels include confounding bias at a moderate risk; bias from participant selection at a moderate risk; intervention classification bias at a moderate risk; low risk for deviations from intended interventions; moderate risk for missing data; moderate risk for outcome measurement bias; and low risk for reported result selection. Figure 2 (Fig. 2) and figure 3 (Fig. 3) illustrate these risk levels.

### Curriculum in the field of ophthalmology

Most ophthalmology courses offered inadequate exposure (43.9%), whereas a marginally smaller percentage provided a satisfactory level (41.4%). A few courses supplied excessive exposure, and others had ambiguous levels (3.8%). Additionally, a small fraction of courses did not offer any exposure (3.6%) [9], [10]. Most facilities addressing eye conditions operate an ophthalmology clinic at a specified rate. The emergency department handles ophthalmic patients at a particular frequency, while the operating room sees them at another rate. Additionally, family medical practices engage with ophthalmic patients at a specific rate. Conversely, several locations do not interact with ophthalmic patients, leading to confusion about this issue in a specified percentage of instances [7], [9], [10]. Most teaching techniques consist of a theoretical lecture, which accounts for 78.7% of the total. Small group discussions comprise 35.2% of the methods, while self-directed learning accounts for 41.6% [7], [9], [10], [11]. Clinical settings, such as clinics, surgery rooms, and emergency departments, make up 21.4% of the teaching methods [9], [10] (see attachment 1 , tab. 2 in supplementary material 4).

### ICO in undergraduate medical education

Medical school education includes various criteria set by the ICO. The research participants were medical students, interns, residents, and medical institutions. The ICO guidelines applied in the medical curriculum are 20-36% [7], [8], [11], [12]. Four studies had no information or data [9], [10], [13], [14].

### The average duration of ophthalmology education in medical school

Three studies [10], [13], [14] reported an average duration of ophthalmology learning that equals or exceeds 3 weeks. Two studies [7] and [9] reported an average duration of 2 weeks. Lastly, three studies [8] and [11], [12] reported an average duration of ophthalmology learning of fewer than 2 weeks, as shown in attachment 1 , tab. 3 in supplementary material 4.

### ICO core clinical exposure areas

**Figure 4:**
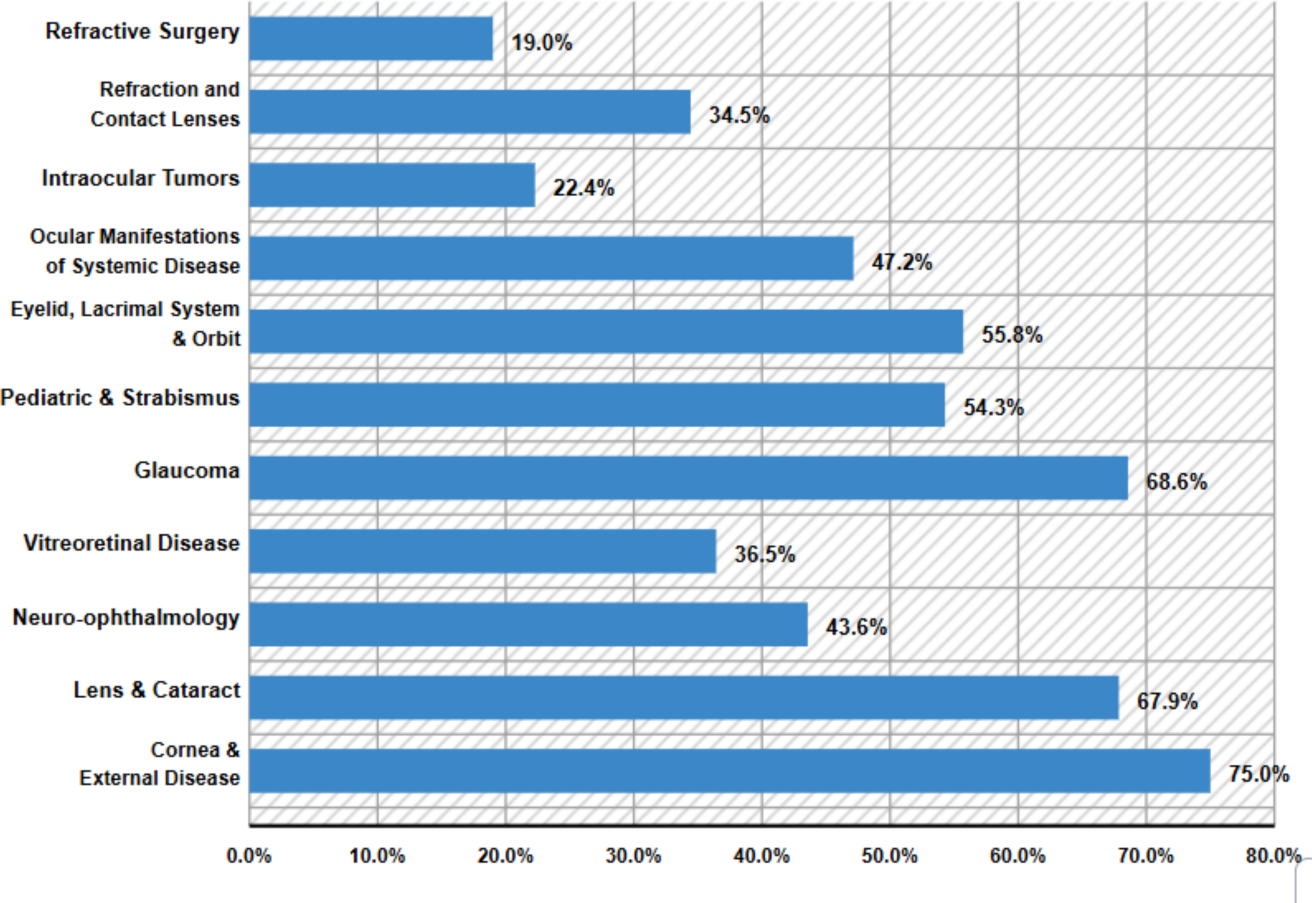
ICO core clinical exposure areas

Most clinical exposure zones in the three studies pertained to corneal or external diseases, representing a significant portion of cases. Specifically, lens and cataract cases comprised 67.9%, neuro-ophthalmology reached 43.6%, and vitreoretinal diseases accounted for 36.5%. Glaucoma made up a notable percentage, alongside other conditions in pediatric ophthalmology, strabismus, and diseases related to the eyelid, lacrimal system, and orbit for 55.8% [7], [11], [14]. Two studies focused on ocular manifestations of systemic diseases, which accounted for 47.2% of cases, intraocular tumors for 22.4%, refraction, and contact lenses for 34.5%, and refractive surgery for 19.0% [7], [11], [14], as depicted in figure 4 (Fig. 4).

### Basic skills of ophthalmology

**Figure 5:**
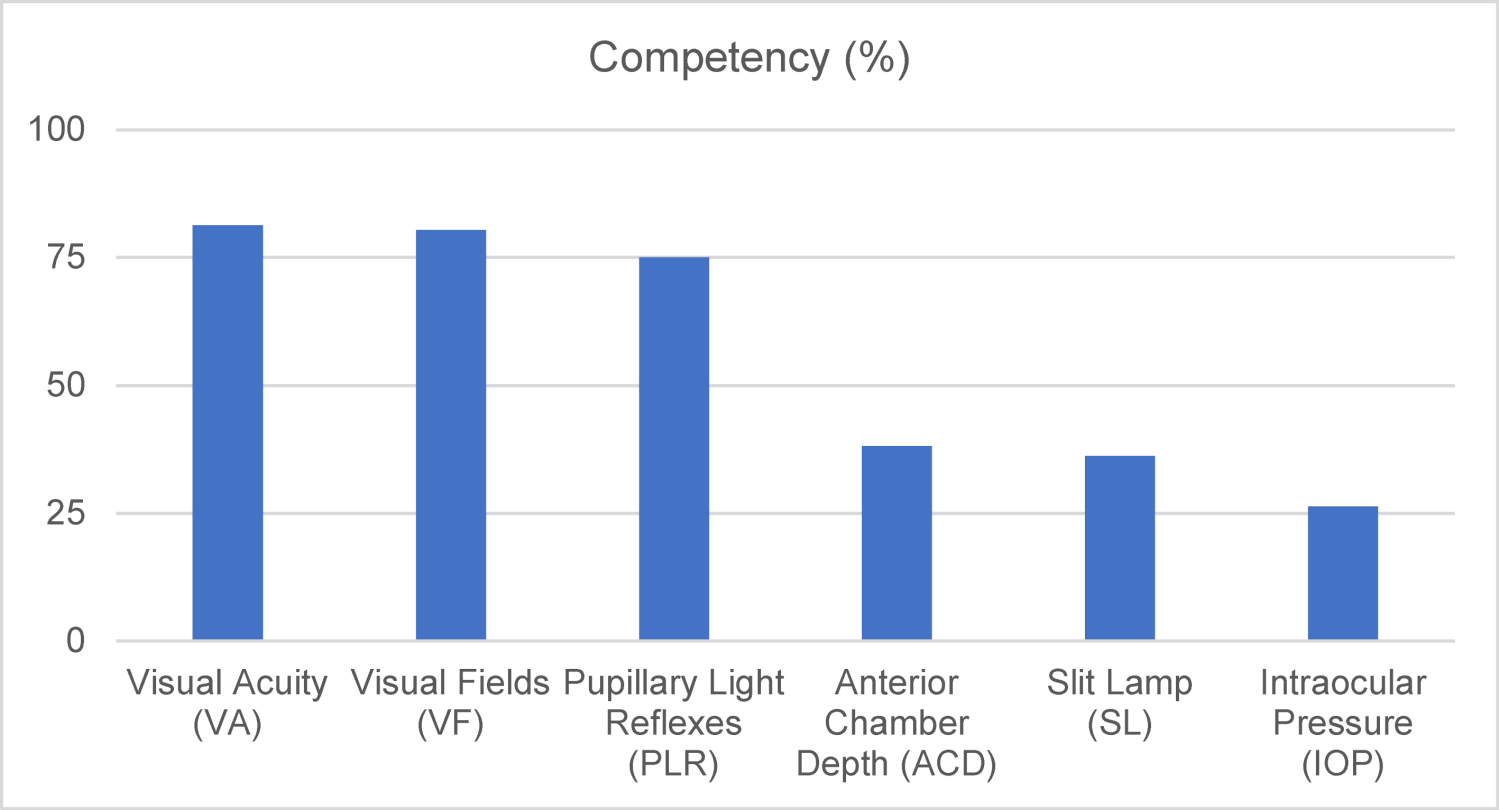
The average poercentage of clinical skills in ophthalmology

Figure 5 (Fig. 5) shows that visual acuity assessment was the predominant clinical skill in three investigations [7], [13], [14], accounting for 81.3% of the cases. The average percentage for visual fields (VF) is 80.4% [10], [14], for pupillary light reflexes is 75.0% [7], [10], for anterior chamber depth is 38.2% [10], [14], for slit lamps is 36.2% [10], [14], and for intraocular pressure (IOP) is 26.3% [7], [10].

The percentage of extraocular muscle involvement is 62.4% direct ophthalmoscope examination, which shows involvement in 35.7% corneal examination with fluorescein, which reveals participation in 26.4%, and 44.1 require referral [10].

## Discussion

### Overview of the available evidence

This systematic review sought to evaluate the current state of undergraduate ophthalmology education in light of the ICO’s recommendations. It comprises a thorough analysis of eight pertinent studies conducted in medical schools involving medical students, interns, and first-year residents. The primary educational goal across most studies was to adhere to the recommendations outlined by the ICO. Ophthalmology courses provide numerous opportunities for students to gain hands-on experience by engaging with ophthalmic patients in various environments, including the ophthalmology clinic, emergency department, operating room, and family medicine practice. Additionally, these courses incorporate a variety of teaching approaches such as theoretical lectures, small group discussions, self-directed learning, and clinical training in both the clinic and operating room, as well as the emergency department [7], [9], [10], [11]. The ICO guidelines suggest a learning period of 40 to 60 hours, translating to five to eight days. Most medical schools provide at least two weeks, often more, for ophthalmology training. However, some programs allocate shorter durations. The ICO highlights key fundamentals and principles of ophthalmology and outlines 11 areas for clinical exposure: cornea and external diseases, lens and cataract, neuro-ophthalmology, vitreoretinal disease, glaucoma, pediatric ophthalmology and strabismus, conditions affecting the eyelids, lacrimal system, and orbit, ocular signs of systemic diseases, intraocular tumors, refraction, contact lenses, and refractive surgery. This study also ranked the clinical exposure areas based on their frequency in curricula. The most common areas were corneal and external diseases, followed by lens and cataracts, while refractive surgery was listed as the least frequent. Additionally, key ophthalmology skills identified in the study included the evaluation of visual acuity as the most common skill, followed by assessing visual fields, pupillary light reflexes, anterior chamber depth, slit lamp examination, and measuring intraocular pressure, which ranked last [7], [10], [13], [14].

The second finding of this study was the prevalence of ICO guideline use, which was 28.15% [7], [8], [11], [12]. The study reveals that the use of international recommendations remains quite limited. Comprehensive guidance from the Royal College of Ophthalmologists and resource sharing between medical schools can help keep ophthalmology integrated into the medical curriculum and improve the quality of undergraduate ophthalmology education. Additionally, this approach can help reduce the workload for local teaching departments and medical schools [11].

### Curriculum development with the ICO guidelines

Standardized medical school curricula guidelines ensure consistency, quality assurance, accreditation, and alignment with practice standards and prepare students for licensure and certification exams. These guidelines help maintain competence among graduates, ensure they achieve quality benchmarks, and equip them for the medical profession. Accreditation is crucial for verifying that institutions uphold excellence standards and effectively prepare students for their careers. By following these guidelines, medical schools can guarantee their graduates deliver high-quality, evidence-based patient care. The ICO has passed a resolution urging all medical schools globally to integrate an ophthalmic education curriculum into their core program instead of treating it as optional. Richard K. took on the role of Chairman of the International Task Force in January 2002 and spearheaded the development of a thorough curriculum for ophthalmology education in medical schools. In 2003, the Chairman interviewed members of the International Federation of Ophthalmological Societies to determine the essential level of ocular knowledge and clinical skills medical graduates should possess [5]. Based on the findings of this study, numerous researchers advocate for including ICO suggestions in the curriculum. Noble et al. identified deficiencies in specific critical areas of undergraduate ophthalmology education in Canada. Implementing a nationwide, uniform curriculum could enhance the competence of medical students in acquiring the necessary ophthalmology knowledge and skills for their future clinical practice [7]. Gostimir et al. suggested that a heightened commitment to the ICO curriculum standards might assist medical students in attaining adequate proficiency in managing patients with eye diseases [8]. Eze et al. suggest that addressing the issues might be achieved by evaluating the curriculum, offering training resources, and following ICO principles [14]. Divya et al. indicated that a comprehensive curriculum assessment, suitable training materials, and targeted teaching strategies for primary care could improve training outcomes [13]. Alselaimy et al. discovered that most Saudi medical schools complied with ICO standards, as many graduates showed proficiency in essential ocular skills. A comprehensive national protocol is necessary to ensure that future general practitioners can effectively care for patients with eye conditions and to develop a premier vision health program in Saudi Arabia. Abuallut et al. propose that creating a competency-based curriculum aligned with ICO guidelines could significantly improve education. Improved training quality and increased exposure would provide students with essential skills, enhance their confidence, and likely increase the ophthalmology workforce [10]. The varied insights gained from this study strongly advocate for medical schools worldwide to adopt the recommendations endorsed by the ICO.

### Strengths and limitations

This study represents the first systematic review and an in-depth evaluation of undergraduate ophthalmology education focusing on ICO standards. The data obtained from all studies’ questionnaires is limited, as a cross-sectional survey approach can shape the undergraduate curriculum. These questionnaires depend solely on self-reporting, which raises the potential for subjective bias from respondents. Furthermore, some studies may face challenges with low response rates. Additionally, several studies do not provide details about clinical exposure areas, essential ophthalmology skills, and how well the program aligns with the guidelines established by the ICO.

### Literature gap and further research

The phrase “literature gap” refers to educational outcomes based on global ophthalmologist recommendations. Future studies should evaluate learning results between groups that followed and those that did not adhere to the ICO guidelines. This assessment will determine whether there are significant differences.

## Conclusion

This study thoroughly evaluated undergraduate ophthalmology education in medical schools, following ICO guidelines. Most research indicates that medical students dedicate the same amount of time to studying ophthalmology, gaining exposure to ICO’s essential clinical areas, and engaging in core clinical activities. However, the use of ICO guidelines is minimal. It is vital to encourage the implementation of these guidelines in medical schools and to conduct further research on the educational outcomes linked to their use implementation.

## Notes

### Data and material availability

Data can be found in the manuscript or supplementary material.

### Author’s ORCID

Luksanaporn Krungkraipetch: [0000-0003-0806-8264]

## Competing interests

The authors declare that they have no competing interests. 

## Supplementary Material

Supplementary material

## Figures and Tables

**Table 1:**
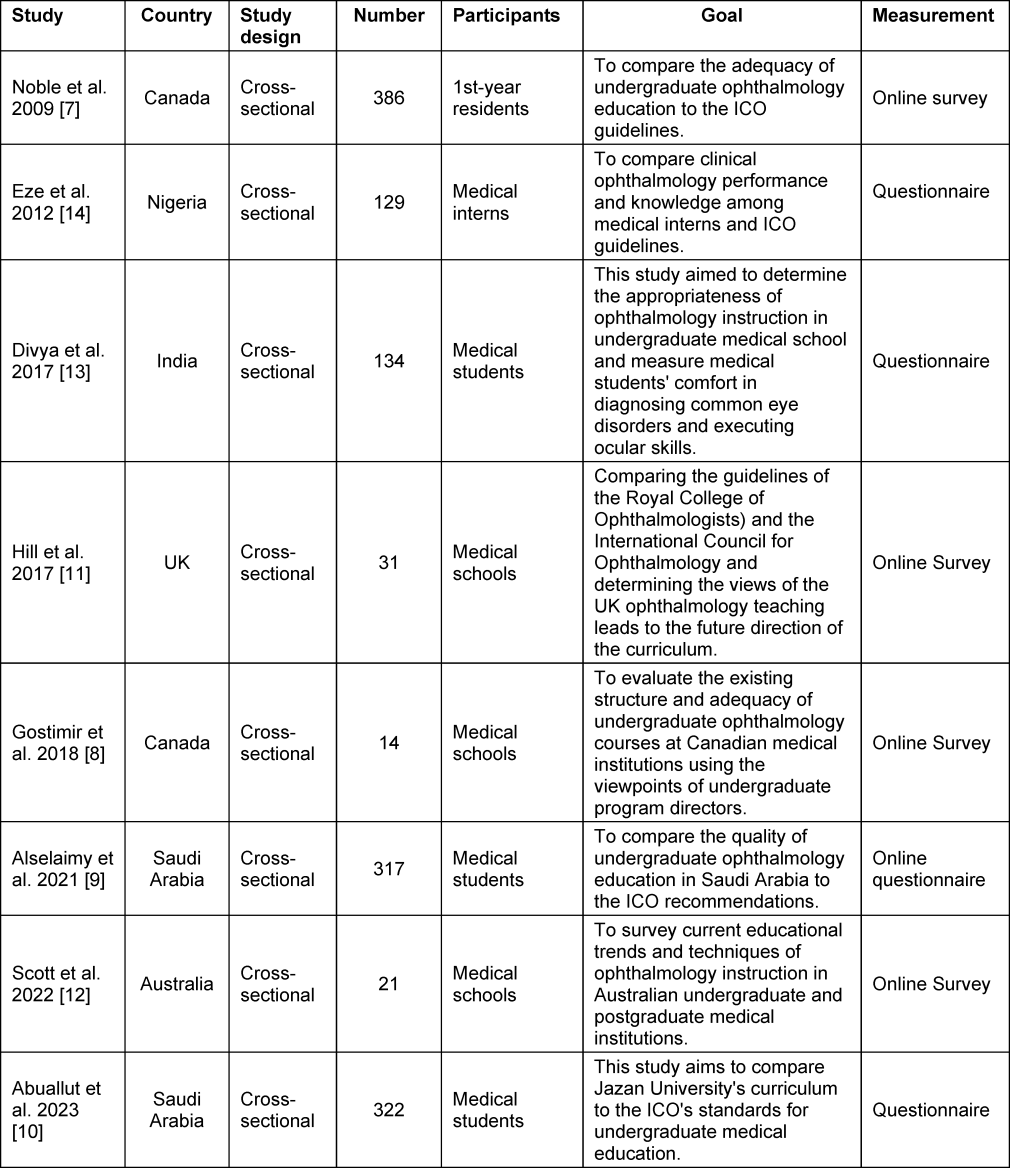
Study characteristics
