# Supplementary material for: Undergraduate ophthalmology education according to International Council of Ophthalmology guidelines: A systematic review
Source: GMS J Med Educ. 2025 Apr 15;42(2):Doc29. doi: 10.3205/zma001753 (PMC12131512; doi:10.3205/zma001753)
Supplement: Supplementary material [file JME-42-29-s-001.pdf]

Attachment 1: Supplementary material

1. Supplementary Material 1: PRISMA checklist and abstract
2. Supplementary Material 2: Full search strategy
3. Supplementary Material 3: Risk of bias score for quantitative impact studies
4. Supplementary material 4: Findings related to primary outcomes, and table 2, 3

1. Supplementary material

a) PRISMA checklist

Topic	No.	Item	Location (pages refer to the article pdf)
**TITLE**			
**Title**	1	Identify the report as a systematic review.	Page 1
**ABSTRACT**			
**Abstract**	2	See the PRISMA 2020 for Abstracts checklist.	Page 1
**INTRODUCTION**			
**Rationale**	3	Describe the rationale for the review in the context of existing knowledge.	Pages 1+ 2
**Objectives**	4	Provide an explicit statement of the objective(s) or question(s) the review addresses.	Pages 1+ 2
**METHODS**			
**Eligibility criteria**	5	Specify the inclusion and exclusion criteria for the review and how studies were grouped for the syntheses.	Page 2
**Information sources**	6	Specify all databases, registers, websites, organisations, reference lists and other sources searched or consulted to identify studies. Specify the date when each source was last searched or consulted.	Page 2
**Search strategy**	7	Present the full search strategies for all databases, registers and websites, including any filters and limits used.	Page 2 and attachment 1, supplementary material 2
**Selection process**	8	Specify the methods used to decide whether a study met the inclusion criteria of the review, including how many reviewers screened each record and each report retrieved, whether they worked independently, and if applicable, details of automation tools used in the process.	Page 2
**Data collection process**	9	Specify the methods used to collect data from reports, including how many reviewers collected data from each report, whether they worked independently, any processes for obtaining or confirming data from study investigators, and if applicable, details of automation tools used in the process.	Page 2

**Data items**	10a	List and define all outcomes for which data were sought. Specify whether all results that were compatible with each outcome domain in each study were sought (e.g. for all measures, time points, analyses), and if not, the methods used to decide which results to collect.	Page 2
	10b	List and define all other variables for which data were sought (e.g. participant and intervention characteristics, funding sources). Describe any assumptions made about any missing or unclear information.	Page 2
**Study risk of bias assessment**	11	Specify the methods used to assess risk of bias in the included studies, including details of the tool(s) used, how many reviewers assessed each study and whether they worked independently, and if applicable, details of automation tools used in the process.	Page 2
**Effect measures**	12	Specify for each outcome the effect measure(s) (e.g. risk ratio, mean difference) used in the synthesis or presentation of results.	Page 2
**Synthesis methods**	13a	Describe the processes used to decide which studies were eligible for each synthesis (e.g. tabulating the study intervention characteristics and comparing against the planned groups for each synthesis (item 5)).	Page 2
	13b	Describe any methods required to prepare the data for presentation or synthesis, such as handling of missing summary statistics, or data conversions.	Page 2
	13c	Describe any methods used to tabulate or visually display results of individual studies and syntheses.	Page 2
	13d	Describe any methods used to synthesize results and provide a rationale for the choice(s). If meta-analysis was performed, describe the model(s), method(s) to identify the presence and extent of statistical heterogeneity, and software package(s) used.	N/A
	13e	Describe any methods used to explore possible causes of heterogeneity among study results (e.g. subgroup analysis, meta-regression).	N/A
	13f	Describe any sensitivity analyses conducted to assess robustness of the synthesized results.	N/A
**Reporting bias assessment**	14	Describe any methods used to assess risk of bias due to missing results in a synthesis (arising from reporting biases).	Page 2
**Certainty assessment**	15	Describe any methods used to assess certainty (or confidence) in the body of evidence for an outcome.	Page 2
**RESULTS**			
**Study selection**	16a	Describe the results of the search and selection process, from the number of records identified in the search to the number of studies included in the review, ideally using a flow diagram.	Page 3 and figure1
	16b	Cite studies that might appear to meet the inclusion criteria, but which were excluded, and explain why they were excluded.	Figure 1

**Study characteristics**	17	Cite each included study and present its characteristics.	Page 3 and table1
**Risk of bias in studies**	18	Present assessments of risk of bias for each included study.	Page 4 and figure 2
**Results of individual studies**	19	For all outcomes, present, for each study: (a) summary statistics for each group (where appropriate) and (b) an effect estimate and its precision (e.g. confidence/credible interval), ideally using structured tables or plots.	Pages 3–7, tables 2, 3, and figures 3, 4 in attachment 1, supplementary material 4
**Results of syntheses**	20a	For each synthesis, briefly summarise the characteristics and risk of bias among contributing studies.	Figure 2
	20b	Present results of all statistical syntheses conducted. If meta-analysis was done, present for each the summary estimate and its precision (e.g. confidence/credible interval) and measures of statistical heterogeneity. If comparing groups, describe the direction of the effect.	N/A
	20c	Present results of all investigations of possible causes of heterogeneity among study results.	N/A
	20d	Present results of all sensitivity analyses conducted to assess the robustness of the synthesized results.	N/A
**Reporting biases**	21	Present assessments of risk of bias due to missing results (arising from reporting biases) for each synthesis assessed.	Attachment 1, supplementary material 3
**Certainty of evidence**	22	Present assessments of certainty (or confidence) in the body of evidence for each outcome assessed.	N/A
**DISCUSSION**			
**Discussion**	23a	Provide a general interpretation of the results in the context of other evidence.	Pages 7–8
	23b	Discuss any limitations of the evidence included in the review.	Pages 7–8
	23c	Discuss any limitations of the review processes used.	Pages 7–8
	23d	Discuss implications of the results for practice, policy, and future research.	Pages 7–8
**OTHER INFORMATION**			
**Registration and protocol**	24a	Provide registration information for the review, including register name and registration number, or state that the review was not registered.	Page 2
	24b	Indicate where the review protocol can be accessed, or state that a protocol was not prepared.	Page 2
	24c	Describe and explain any amendments to information provided at registration or in the protocol.	Attachment 1, supplementary 2

**Support**	25	Describe sources of financial or non-financial support for the review, and the role of the funders or sponsors in the review.	Page 2
**Competing interests**	26	Declare any competing interests of review authors.	Page 8
**Availability of data, code, and other materials**	27	Report which of the following are publicly available and where they can be found: template data collection forms; data extracted from included studies; data used for all analyses; analytic code; any other materials used in the review.	Page 8

N/A, not available

b) PRISMA abstract checklist

Topic	No.	Item	Reported?
**TITLE**			
**Title**	1	Identify the report as a systematic review.	Yes
**BACK-GROUND**			
**Objectives**	2	Provide an explicit statement of the main objective(s) or question(s) the review addresses.	Yes
**METHODS**			
**Eligibility criteria**	3	Specify the inclusion and exclusion criteria for the review.	Yes
**Information sources**	4	Specify the information sources (e.g. databases, registers) used to identify studies and the date when each was last searched.	Yes
**Risk of bias**	5	Specify the methods used to assess risk of bias in the included studies.	Yes
**Synthesis of results**	6	Specify the methods used to present and synthesize results.	Yes
**RESULTS**			
**Included studies**	7	Give the total number of included studies and participants and summarise relevant characteristics of studies.	Yes
**Synthesis of results**	8	Present results for main outcomes, preferably indicating the number of included studies and participants for each. If meta-analysis was done, report the summary estimate and confidence/credible interval. If comparing groups, indicate the direction of the effect (i.e. which group is favoured).	Yes
**DISCUSSION**			
**Limitations of evidence**	9	Provide a brief summary of the limitations of the evidence included in the review (e.g. study risk of bias, inconsistency and imprecision).	Yes
**Interpretation**	10	Provide a general interpretation of the results and important implications.	Yes
**OTHER**			
**Funding**	11	Specify the primary source of funding for the review.	No
**Registration**	12	Provide the register name and registration number.	Yes

Page MJ, McKenzie JE, Bossuyt PM, Boutron I, Hoffmann TC, Mulrow CD, et al. The PRISMA 2020 statement: an updated guideline for reporting systematic reviews. MetaArXiv. 2020, September 14. DOI: 10.31222/osf.io/v7gm2
For more information, visit: https://www.prisma-statement.org

2. Supplementary material 2: Full search strategy

OBJECTIVES:

To survey undergraduate ophthalmology education based on the International Council of Ophthalmology recommendations.

SCOPE

The scope of the review is listed below, outlined according to the PICO (Participants, Intervention, Comparators, Outcome) framework:	
**Participants:**	Medical schools/Medical students/Medical interns/1st year residents
**Intervention:**	International Council of Ophthalmology guidelines
**Comparator(s)**	Non-ICO guidelines
**Outcome:**	
**Primary Outcome Measure**	International Council of Ophthalmology guidelines in undergraduate medical education
**Secondary Outcome Measure**	Prevalence of ICO guidelines used in medical curriculum

METHODS:

Study eligibility

**Inclusion Criteria:**	
**Study Characteristics**	Empirical research studies (cross-sectional)
	Global geography setting
	Years of analysis between 2000-2024
**Report Characteristics**	Published between 2000-2024
	Published in English
**Exclusion Criteria:**	Non-full paper with peer review
	Non-empirical (e.g., viewpoint, opinion, commentary)

Information sources

**Electronic Databases**	PubMed<br>Cochrane<br>Scopus<br>ERIC
**Additional Sources**	Cited Reference in articles identified via electronic database searches.
**Search Terms**	"Medical education"<br>"Ophthalmology"<br>"Undergraduate"<br>"International council ophthalmology guidelines"

a) PubMed search strategy

#1	education, medical [MeSH Terms]
#2	academic, medical centers [MeSH Terms]
#3	schools, medical [MeSH Terms]
#4	students, medical [MeSH Terms]
#5	medical education [Title/Abstract]
#6	medical school [Title/Abstract]
#7	medical student [Title/Abstract]
#8	education, medical, undergraduate [MeSH Terms]
#9	undergraduate med*[Title/Abstract]
#10	undergraduate stud*[Title/Abstract]
#11	ophthalmology [MeSH Terms]
#12	ophthalmology [Title/Abstract]
#13	#1 OR #2 OR #3 OR #4 OR #5 OR #6 OR #7 OR #8 OR #9 OR #10 OR #11 OR #12
#14	international council [Title/Abstract]
#15	international council ophthalmology [Title/Abstract]
#16	guidelines as topic [MeSH Terms]
#17	international council ophthalmology guidelines [Title/Abstract]
#18	#14 OR #15 OR # 16 OR # 17
#19	#13 OR #19 with Publication Year from 2000 to 2024

b) Cochrane Central Register of Controlled Trials (CENTRAL) search strategy

#1	MeSH descriptor: [Education, Medical] explode all trees
#2	MeSH descriptor: [Academic Medical Centers] explode all trees
#3	MeSH descriptor: [Schools, Medical] explode all trees
#4	MeSH descriptor: [Students, Medical] explode all trees
#5	(medical education):ti,ab,kw
#6	(medical school):ti,ab,kw
#7	(medical student):ti,ab,kw
#8	MeSH descriptor: [Education, Medical, Graduate] explode all trees
#9	(undergraduate med*):ti,ab,kw
#10	(undergraduate stud*):ti,ab,kw
#11	MeSH descriptor: [Ophthalmology] explode all trees
#12	(ophthalmology):ti,ab,kw
#13	#1 OR #2 OR #3 OR #4 OR #5 OR #6 OR #7 OR #8 OR #9 OR #10 OR #11 OR #12
#14	(international council):ti,ab,kw
#15	(international council ophthalmology):ti,ab,kw
#16	MeSH descriptor: [Guidelines as topic] explode all trees
#17	(international council ophthalmology guidelines):ti,ab,kw
#18	#14 OR #15 OR # 16 OR # 17
#19	#13 OR #18 with Publication Year from 2000 to 2024

c) Education Resources Information Center (ERIC) search strategy

1	medical education
2	undergraduate
3	ophthalmology
4	international council ophthalmology
5	international council ophthalmology guideline
6	medical education AND undergraduate
7	medical education AND undergraduate AND ophthalmology

d) Scopus

1	Medical education
2	Undergraduate
3	Ophthalmology
4	International Council Ophthalmology
5	International Council Ophthalmology Guideline
6	Medical education AND Undergraduate
7	Medical education AND Undergraduate AND Ophthalmology

3. Supplementary material 3: Risk of bias score for quantitative impacts studies

1st Author Last name	Bias due to con-founding	Bias in selection of participants into study	Bias in classification of intervention	Bias due to deviations from intended interventions	Bias due to missing data	Bias in measurement of outcomes	Bias in selection of the reported result	Overall risk of bias score
Noble	2	1	0	0	1	1	0	1
Eze	1	0	0	0	1	1	0	0
Divya	1	0	0	0	0	1	0	0
Hill	2	0	1	0	1	1	0	1
Gostimir	2	2	1	0	1	1	0	1
Alselaimy	1	0	0	0	0	1	0	0
Scott	1	0	0	0	1	1	0	0
Abuallut	2	0	0	0	1	1	0	1

Sterne JA, Hernan MA, Reeves BC, Savovic J, Berkman ND, Viswanathan M, et al. ROBINS-I: a tool for assessing risk of bias in non-randomised studies of interventions. BMJ 2016;355: i4919. DOI: 10.1136/bmj. i4919

Key

0	Low risk of bias
1	Moderate risk of bias
2	Serious risk of bias
3	Critical risk of bias
NI	No information

4. Supplementary material 4: Findings related to primary outcomes, and table 2, 3

a) Findings related to primary outcomes

Study	Detail
Noble et al., 2009 [7]	The vast majority (76.2%) of respondents reported having had little more than one week of overall exposure to ophthalmology. Several ICO key subspecialty topics were adequately covered, including lens/cataract (81.1%) and cornea/external diseases (81.6%); nevertheless, certain areas, including vitreoretinal disease (41.9%), did not receive appropriate time allocation. Similarly, competency was achieved in some ICO examination abilities, such as visual acuity assessment (83.3%) and pupillary reflexes (90.7%), but not in others, including as fundoscopy (52.3%), slit-lamp examination (44.8%), and intraocular pressure assessment (19.9%). When asked if they had acquired sufficient ophthalmic knowledge and skills throughout medical school, just 42.9% and 25.9% agreed, respectively.
Eze et al., 2012 [14]	The response rate was 88.7%. The duration of undergraduate ophthalmology exposure varied from one to four weeks. Exposure was frequent enough in cornea/external eye (95.3%), lens/cataract (95.3%), and glaucoma (92.2%), but not in vitreoretinal disease (47.3%), neuro-ophthalmology (45.7%), or refractive surgery (0.0). The majority were proficient in visual acuity testing (97.7%) and visual field examination (93.0%). There was less competency in anterior chamber assessment (49.6%) and slit-lamp examination (39.5%). The majority could accurately diagnose conjunctivitis (96.1%) and cataracts (90.7%), but not strabismus (42.6%) or macular degeneration (20.2%).
Divya et al., 2017 [13]	In all, 134 students took part in the research. They had received classroom-based teaching for an average of 96.2 ± 5.9 hours and clinic-based instruction for an average of 112.5 ± 11.3 hours. When it came to cataracts and eyelid abnormalities, the participants felt comfortable enough to diagnose eye problems, but not when it came to ophthalmic crises. Only 45.5% have adequate information about community ophthalmology. Direct ophthalmoscopy (41%), in contrast to pupillary response assessment (80.6%) and visual acuity testing (93.3%), demonstrated lower levels of proficiency among respondents.
Hill et al., 2017 [11]	A response rate of 93% was obtained. The information and clinical skills provided in UK medical schools align with the RCOphth requirements but do not meet them. UK medical schools use a varied range of assessment methodologies during ophthalmology rotations. Variation was also seen in the organization and methods of ophthalmology education. However, teaching leads noted a considerable unanimity on the curriculum's future path.
Gostimir et al., 2018 [8]	Responses were received from 7 of 14 (50%) program directors. All the responses represented metropolitan institutions with over 100 seats. After merging survey and website data, only 5 of 14 (35.7%) schools required a clinical clerkship in ophthalmology. In all cases, the obligatory rotation is fewer than two weeks. Groups.
Alselaimy et al., 2021 [9]	The study included 317 individuals from various Saudi medical schools. Our study results followed the ICO guidelines in several ways, including ophthalmology training during medical school (93.4%), a 2-week ophthalmology course (56.2%), necessary knowledge for patient referral (55.8%), competency in most basic ophthalmic skills, and participants receiving different teaching methods (theoretical lectures and clinical settings).
Scott et al., 2022 [12]	A total response rate of 90.48% (19 of 21 medical schools) was received, with strong representation across Australia. Ophthalmology rotations were necessary in 63.3% of cases (n = 12), while 36.7% (n = 7) did not have mandated periods. This is comparable to the USA (16%), Canada (35.7%), and the UK (65%). 74% (n = 14) say ophthalmology is not a priority in their curriculum. All respondents reported students having at least one clinical day in ophthalmology, with total instruction time ranging from fewer than six hours (36.9%) to more than two weeks (10.5%).
Abuallut et al., 2023 [10]	Among the respondents, 31 (9.6%) reported no ophthalmology experience, whereas 117 (36.3%) reported insufficient exposure. A significant proportion of participants demonstrated competency in a variety of areas, including acquiring an eye history (n = 113, 35.1%), measuring visual acuity (n = 201, 62.4%), and analyzing extraocular movement. In total, 98 (30.4%) of the participants exhibited an interest in ophthalmology, while the majority (n = 224, 69.6%) did not.

b) Table 2: Ophthalmology curricula

**Ophthalmology Curricula**	**N (%)**
**Ophthalmology course exposure (2 studies)**	
No exposure	23 (3.60)
Too little exposure	279 (43.6)
Right amount of exposure	243 (41.4)
Too much exposure	27 (4.2)
Unsure	24 (3.8)
**Location encountering ophthalmic patients (3 studies)**	
Ophthalmology clinic	369 (87.1)
Emergency department	102 (23.9)
Operation room	55 (11.4)
Family medicine practice	91 (17.3)
No contact with ophthalmic patients	290 (45.3)
**Teaching method (4 studies)**	
Theoretical lectures	786 (78.7.0)
Clinical (2 studies)<br>(clinic, operation room, emergency department)	136 (21.4)
Small group discussion	226 (35.2)
Self-directed learning	322 (41.6)

c) Table 3: Average time spent learning ophthalmology in medical schools

Study	Average time (weeks)					
	<1	1	1–2	2	2–3	>3
Noble et al., 2009				/		
Eze et al., 2012						/
Divya et al., 2017						/
Hill et al., 2017			/			
Gostimir et al., 2018			/			
Alselaimy et al., 2021				/		
Scott et al., 2022			/			
Abuallut et al., 2023						/
